# Ipatasertib, an oral AKT inhibitor, in combination with carboplatin exhibits anti-proliferative effects in uterine serous carcinoma

**DOI:** 10.1080/07853890.2023.2177883

**Published:** 2023-02-11

**Authors:** Wesley C. Burkett, Ziyi Zhao, Meredith A. Newton, Wenchuan Sun, Boer Deng, Angeles Alvarez Secord, Chunxiao Zhou, Victoria Bae-Jump

**Affiliations:** a Division of Gynecologic Oncology, Department of Obstetrics and Gynecology, University of North Carolina at Chapel Hill, NC; b Department of Gynecologic Oncology, Beijing Obstetrics and Gynecology Hospital, Capital Medical University, Beijing Maternal and Child Health care Hospital, Beijing, P. R. China; c Division of Gynecologic Oncology, Department of Obstetrics and Gynecology, Duke University, Durham, NC; d Lineberger Comprehensive Cancer Center, University of North Carolina at Chapel Hill, NC

**Keywords:** Ipatasertib, apoptosis, invasion, synergy, carboplatin, uterine serous carcinoma

## Abstract

**Purpose:**

Uterine serous carcinoma (USC) exhibits worse survival rates compared to the endometrioid subtype, and there is currently no effective treatment options for recurrence of this disease after platinum-based chemotherapy. Activation of PIK3CA/AKT/mTOR signaling pathway is a common biological feature in USC.

**Materials and Methods:**

Ipatasertib (IPAT) is an investigational, orally administered, ATP-competitive, highly selective inhibitor of pan AKT that has demonstrated anti-proliferative activity in a variety of tumor cells and tumor models. In this study, we used IPAT, carboplatin and their combination to investigate the anti-tumor activity in SPEC-2 and ARK-1 cells.

**Results:**

Our results indicate that IPAT combined with carboplatin at low doses was more effective at reducing proliferation, inducing apoptosis and causing cellular stress than IPAT or carboplatin alone. In particular, inhibition of the PIK3CA/AKT/mTOR pathway and induction of DNA damage were involved in the synergistic inhibition by combination treatment of cell viability in USC cells treated with the combination. Furthermore, IPAT in combination with carboplatin significantly reduced cell adhesion and inhibited cell invasion.

**Conclusions:**

These findings suggest that the combination of IPAT and carboplatin has potential clinical implications for developing new USC treatment strategies.

## Introduction

Endometrial cancer (EC) is the most common gynecologic malignancy in the United States and its incidence is increasing [1]. In addition to an increasing incidence, EC mortality has increased and is now comparable to that of ovarian cancer [2]. Non-endometrioid subtypes of EC exhibit worse survival rates compared to the endometrioid subtype and are significantly contributing to the rapid rise in the incidence of EC [3]. More than half of patients who are diagnosed with uterine serous carcinoma (USC), the most common non-endometrioid EC, present with metastatic disease [4,5]. Platinum-based chemotherapy is the backbone of systemic treatment for advanced-stage EC [6–8]. However, limited treatment options are currently available for disease progression following platinum-based chemotherapy with median overall survival ranging from 12 to 18 months [9,10]. Therefore, additional active therapies are desperately needed.

Although p53 mutations are one of the most common somatic mutations in USC, the amplification and mutation of ErbB2, an upstream regulator of the PIK3CA/AKT/mTOR-signaling pathway, and PIK3CA have recently been identified as other common genetic defects [11,12]. Alterations in ErbB2 and PIK3CA are major factors in activating the PIK3CA/AKT/mTOR pathway, accounting for about 80% of USC cases [11]. Hyperactivation of the PI3K/AKT/mTOR signaling pathway has previously been reported to be central to the control of cell viability, apoptosis, cell cycle, metabolism, and chemoresistance in multiple human cancers, including USC. Thus, these special mutational patterns and functional alterations may contribute to the development of specific molecular targets for USC [13–16]. Ipatasertib (IPAT) is an investigational, orally administered, ATP-competitive, highly selective inhibitor of pan AKT that has demonstrated anti-proliferative activity in pre-clinical models including prostate, breast, and ovarian cancers [17,18]. IPAT is well tolerated and safe in combination with chemotherapy or hormone therapy as demonstrated in a first-in-human phase I clinical trial [19]. Multiple phase II and III clinical trials of IPAT have shown anti-tumor activity in metastatic prostate cancer and longer progression-free survival in triple-negative breast cancer [20–22]. Currently, there are no dedicated trials investigating IPAT in EC and trials of other AKT inhibitors in EC are limited [23]. Given these findings, we evaluated the anti-proliferative efficacy of IPAT and its synergistic effect in combination with standard-of-care carboplatin on cell proliferation in human USC cell lines.

## Materials and methods

### Cell culture and reagents

The human USC cell lines ARK-1 with a *PIK3CA* gene mutation and SPEC-2 with a PTEN deficiency were used for experiments [24,25]. The ARK-1 cells were cultured in RPMI 1640 medium with 10% fetal bovine serum (FBS). The SPEC-2 cells were cultured in DMEM/F12 medium with 10% FBS. Cells were cultured in humidified 5% CO_2_ at 37 °C. All media included 100 units/mL penicillin and 100 µg/mL streptomycin. IPAT was provided by Genentech (South San Francisco, CA). Carboplatin was purchased from Sigma (St. Louis, MO). Antibodies were purchased from Cell Signaling Technology (Beverly, MA). All other chemicals were purchased from Sigma-Aldrich (St. Louis, MO).

### MTT assay

Cell proliferation was assessed in ARK-1 and SPEC-2 by MTT assay. Briefly, cells (4000–6000/well) were seeded in 96-well plates and cultured at 37 °C in 5% CO2 overnight and then treated with the indicated doses of IPAT and carboplatin as single agents or in combination for 72 h. 100 ul of MTT solution (5 mg/ml) was added to each well and incubated for another 2 h. The supernatants were aspirated and 100 µl of DOMSO was added to each well to lyse the formazan crystal. Optical density was measured at a wavelength of 570 nm with a Tecan microplate reader (Morrisville, NC). The effect of IPAT and carboplatin on proliferation was assessed as the percentage of inhibition compared to untreated cells. IC50 calculator (AAT Bioquest; Sunnyvale, CA) was used to detect IC50 for IPAT and carboplatin. Bliss Independence model was used to determine whether drug effects are additive (CI = 1), synergistic (CI < 1) or antagonistic (CI > 1) [26]. Results represent the median of three separate experiments, each performed in quadruplicate.

### Colony assay

The ARK-1 and SPEC-2 cells were plated overnight in 6-well plates at a density of 400 cells/well and exposed to indicated concentrations of IPAT and carboplatin for 48 h. After treatment, cells were washed with PBS and fresh media was added to each well, with medium changes every three days. After 2 weeks of treatment, cells were fixed in 100% methanol for 15 min and stained 0.5% crystal violet for 10 min. Colonies were captured and counted under a microscope.

### Cleaved caspase-3 ELISA assay

The ARK-1 and SPEC-2 cell lines were treated with IPAT (1 µM), carboplatin (10 µM) or a combination of IPAT 1 µM and carboplatin 10 µM in 6-well plates for 14 h at 37 °C. After replacing the media with 1X caspase lysis buffer, cell lysates were collected and incubated with reaction buffer containing 200 µM of caspase 3 substrates (AAT Bioquest) for 30 min. The Fluorescence was measured at Ex/Em= 400/505 nm using a Tecan plate reader. Experiments were repeated three times in triplicate.

### Reactive oxygen species (ROS) assay

The DCFDA assay was used to quantify intracellular ROS generation. The ARK1 and SPEC-2 cells (8000–12,000 cells/well) were seeded in 96-well plates overnight and then treated with IPAT 1 µM, carboplatin 10 µM, and a combination of IPAT 1 µM and carboplatin 10 µM for 16 h. 20 ul of 10 uM DCFDA were added to each well and incubated for 20 min at 37 °C in the dark. Fluorescence intensity was recorded at an excitation wavelength of 485 nm and emission at 526 nm with a Teca plate reader. The same plate was then used to add 10 ul of MTT solution (5 mg/ml) to each well. MTT results were used to normalize fluorescence measurements on the same plate.

### JC-1 assay

The loss of mitochondrial membrane potential was analyzed in ARK-1 and SPEC-2 cells using the fluorescent cationic dye JC-1 (AAT Bioquest). Cells were seed in a 96-well plate overnight and then treated with IPAT 1 µM, carboplatin 10 µM, and a combination of IPAT 1 µM and carboplatin 10 µM for 8 h. 1 µl of JC-1 (200 µM) was added to each well and incubated the plate for 30 min at 37 °C in a dark. The levels of JC-1 monomers were detected at an excitation wavelength of 535 nm and an emission wavelength of 590 nm with a Tecan plate reader. Experiments were performed in triplicate and repeated three times to assess consistency.

### TMRE assay

The ARK-1 and SPEC-2 cells were seeded at a density of 2x10^5^ in black 96‐well microplates overnight. The cells were treated with IPAT, carboplatin and a combination at indicated doses for 10 h and then incubated with 1000 µM TMRE (tetramethylrhodamine ethyl ester) for 30 min at 37 °C. After washing the plates with PBS, the plates were measured using a Tecan plate reader at Ex/Em = 549/575 nm.

### Adhesion assay

The ARK-1 and SPEC-2 cells (2500/well) were seeded in triplicate in 96-well plates coated with laminin-1 (Sigma-Aldrich) and treated with IPAT, carboplatin and the combination for 2 h at 37 °C. The floating cells have aspired and the adherent cells were fixed using 5% glutaraldehyde for 30 min at room temperature. Plates were washed twice with cold PBS and stained the cells were with 0.1% crystal violet solution for 30 min. 100 ul of 10% acetic acid was added to each well to solubilize the dye. Optical density of each well was recorded at 575 nm using a Tecan microplate reader.

### Wound healing assay

Wound healing assay was used to evaluate the capacity for migration in ARK-1 and SPEC-2 cells. The cells (2–3 × 105 cells/well) were plated in 6-well plates for 24 h. When cells grew to > 80% confluence under microscopy, wounds were scratched in a straight line with a 200 ul sterile pipette tip. After washing twice with PBS in each well, cells were treated with IPAT, carboplatin and the combination of IPAT and carboplatin for 24–72 h. The cells in each well were photographed using a microscope after 24, 48 and 72 h of treatment. Cell migration capacity was analyzed by ImageJ (National Institutes of Health; Bethesda, MD).

### Western immunoblotting

The ARK-1 and SPEC-2 cells (2–3 x 10^5^ cells/well) were plated in 6-well plates and cultured overnight followed by drug treatments with the indicated drug for 12–24 h. Cells were disrupted in ice-cold RIPA lysis buffer, and the supernatant was clarified by centrifugation at 12,000 rpm for 20 min and stored at −80 °C. Protein concentration was determined by a BCA protein assay kit (Thermo Fisher Scientific). Protein extracts were separated by an SDS-polyacrylamide gel electrophoresis, and transferred to an equilibrated polyvinylidene difluoride (PVDF) membrane 4 °C. The membranes were blocked with 5% fat-free milk for 1 h at room temperature and immunoblotted with the indicated antibodies. Target protein bands were visualized and quantified using an enhanced chemiluminescence kit (Thermo Fisher Scientific) on the ChemiDoc Image System (Bio-Rad; Hercules, CA). Experiments were performed in duplicate to assess for consistency.

### Statistical analysis

Data are given as the mean ± SD. Statistical significance was analyzed by the two-sided unpaired Student’s *t*-test from at least 3 replicates. GraphPad Prism 6 (La Jolla, CA) was used for all graphs and significance tests. *p* values of <0.05 were considered to have significant group differences.

## Results

### IPAT in combination with carboplatin inhibits cell proliferation

**Figure 1.**
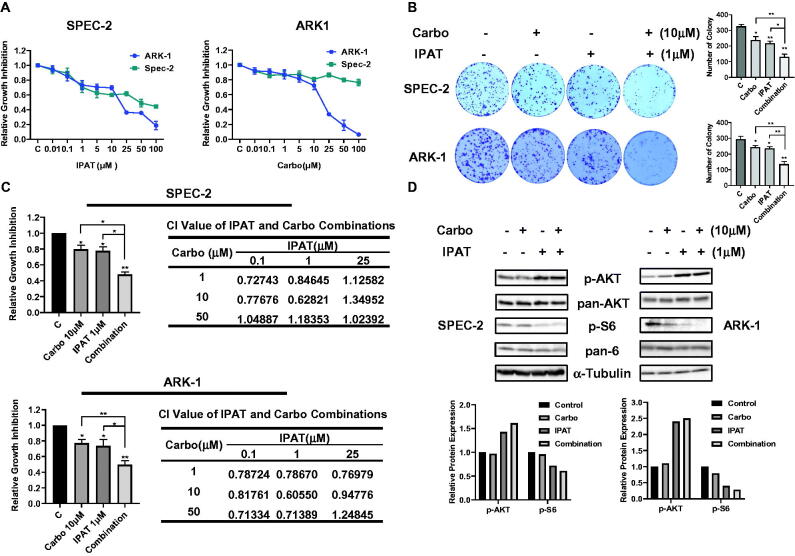
IPAT in combination with carboplatin inhibits cell proliferation. The SPEC-2 and ARK1 cells were treated with different concentrations of IPAT and carboplatin for 72 hours. Cell proliferation was evaluated by MTT assay, as described in Materials and Methods (A). SPEC-2 and ARK1 cells were treated with IPAT (1 uM), carboplatin (10 uM) and the combination treatment for 48 hours, and then the cells continued to culture for 2 weeks, followed by colony assay (B). Combination treatment of IPAT and carboplatin at low doses for 72 hours showed synergic inhibitory effects on cell proliferation in both cell lines. Combination index (CI) was determined using The Bliss Independence model (CI < 1, synergistic effect; CI = 1, additive effect; CI > 1, antagonistic effect). Results represent the median of three separate experiments, each performed in quadruplicate (C). The effects of IPAT, carboplatin and the combination on phosphorylated AKT and phosphorylated S6 were assessed by western blotting (D). * *p* < 0.05, ** *p* < 0.01.

To evaluate the antiproliferative effects of IPAT and carboplatin as single agents, we used ARK-1 and SPEC-2 cells treated with different concentrations of IPAT, carboplatin and a combination of IPAT and carboplatin for 72 h. Cell proliferation was measured with MTT assay. Figure 1(A) demonstrates the inhibition of cell proliferation in a dose-dependent manner in both cell lines when treated with IPAT and carboplatin. Mean IC50 values for IPAT in ARK-1 and SPEC-2 cells were 21.58 and 23.33 uM, respectively, and the IC50 values of carboplatin in ARK1 and SPEC-2 were 18.769 and >100 uM, respectively. Treating SPEC-2 cells with different concentrations of carboplatin for 72 h did not achieve an IC50 value of inhibition, suggesting that SPEC-2 cells are insensitive to carboplatin compared to ARK-1 cells. Given that colony formation assay is a measure of long-term tumor cell survival *in vitro* and an important characteristic of tumor growth *in vivo*, the effect of IPAT, carboplatin and the combination of clonogenicity in ARK-1 and SPEC-2 cells were evaluated. 10 µM carboplatin and 1 µM IPAT treatments reduced the colony formation by 16.8% and 19.5% in ARK-1 cells, and 21.8% and 26.1% in SPEC-2 cells Furthermore, a significant reduction in colony formation was observed when 1 µM IPAT was combined with 10 µM carboplatin compared to single agent treatment in both cell lines (Figure 1(B)). To evaluate the synergistic effect of the combination of IPAT and carboplatin in ARK-1 and SPEC-2 cells, the Bliss Independence model was used to calculate the Combination Index (CI) for each combination treatment based on the amount of cell viability in each combination treatment group. We first selected three doses of each drug based on the inhibitory concentration, with dose ranges of IC15, IC30 and IC50 for IPAT and IC15, IC30 and IC60 for carboplatin, respectively, and then three different doses of the two drugs were combined to treat SPEC-2 and ARK-1 cells for 72 h. MTT assays showed that the combination of low-doses of IPAT and carboplatin exhibited more potent effects in inhibiting cell proliferation in both cell lines than a single agent. In ARK1 cells, the combination of 1 or 10 uM IPAT and high-dose carboplatin also significantly inhibited cell proliferation compared with IPAT, carboplatin, or the control (CI < 1). These results indicate that the combination of IPAT and carboplatin at low doses produced synergetic effects in growth inhibition in both the ARK-1 and SPEC-2 cells (Figure 1(C)).

Considering the role of PI3K/AKT pathway inhibition by IPAT, phosphorylation of AKT (Ser473) and S6 (Ser235/236) was assessed by western blotting after 24 h of treatment with IPAT, carboplatin and the combination in both cell lines. Consistent with other studies, the results showed that IPAT increased the expression of phosphorylated AKT and decreased phosphorylated S6 expression. Increased expression of phosphorylated AKT is associated with IPAT competing with ATP for binding sites and preventing dephosphorylation of the p-AKT complex [18]. Combination treatment did not alter the IPAT-mediated effects on AKT and S6 phosphorylation in either cell line, indicating that the inhibitory effect of the combination treatment depends on the inhibition of PI3K/AKT pathway (Figure 1(D)).

### Effect of IPAT in combination with carboplatin on the induction of apoptosis

**Figure 2.**
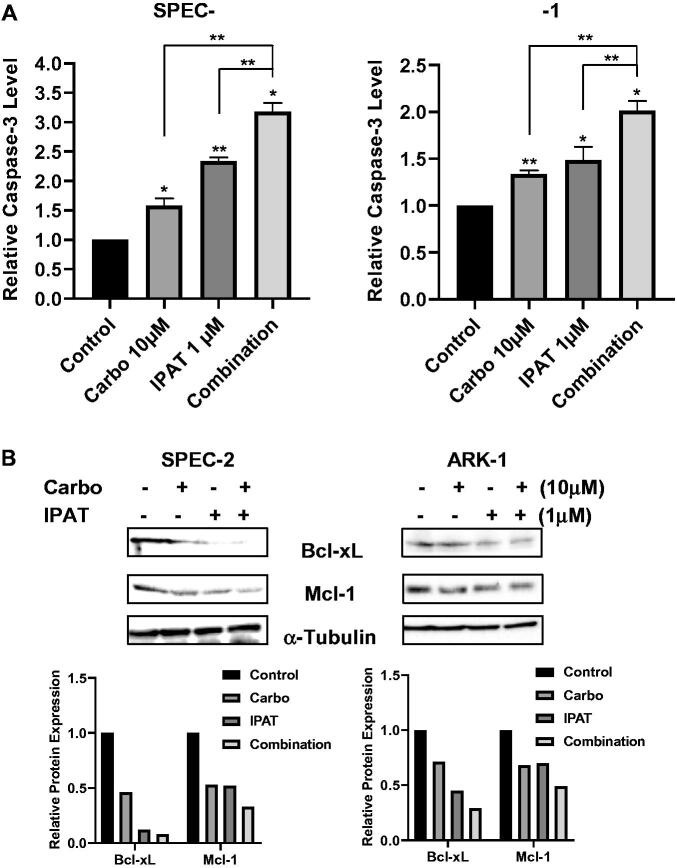
IPAT in combination with carboplatin induces apoptosis. Cleaved caspase 3 assay was performed on SPEC-2 and ARK1 cells after 14 hours of treatment with IPAT, carboplatin and the combination (A). Western blotting analysis of BCL-XL and MCL-1 was performed on lysates from SPEC-2 and ARK1 cells after 24 hours of treatments. α-Tubulin included as a loading control (B). Values in the bar graphs represent the mean ± SD of at least three independent experiments. * p < 0.05, ** p < 0.01.

To analyze the induction of apoptosis in ARK-1 and SPEC-2 cell lines treated with IPAT, carboplatin and the combination, cleaved caspase-3 assays showed that treating ARK-1 and SPEC-2 cells with IPAT and carboplatin demonstrated significant induction in the activity of cleaved caspase-3 with the greatest effect seen when treating with the drug combination (Figure 2(A)). In SPEC-2 and ARK-1 cells, 10 µM carboplatin induced cleaved caspase-3 by 1.57- and 1.33-fold, respectively, while 1 µM IPAT increased cleaved caspase 3 activity by 2.42- and 1.49-fold, respectively. Combination of 1 µM IPAT and 10 µM carboplatin was significantly more effective in inducing cleaved caspase-3 activity and produced 3.28- and 2.01-fold induction in SPEC-2 and ARK-1 cells, respectively. Western immunoblotting was performed to assess the expression of the anti-apoptotic proteins Bcl-XL and MCL-1 when treating ARK-1 and SPEC-2 cells with IPAT and carboplatin for 24 h of treatment (Figure 2(B)). The results showed that the reduced expression of both Bcl-XL and MCL-1 when treated with IPAT and carboplatin with the greatest effect seen in combination treatments compared with either IPAT or carboplatin alone in both cell lines.

### Effect of IPAT in combination with carboplatin on the induction of cellular stress

**Figure 3.**
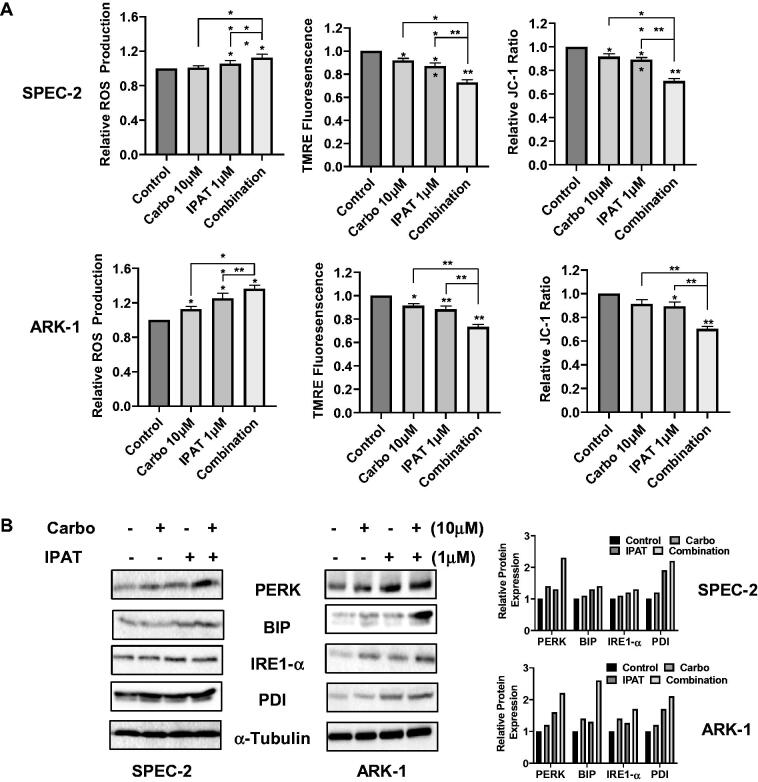
IPAT in combination with carboplatin causes cellular stress. SPEC-2 and Ark1 cells were treated with IPAT, carboplatin and combination for 16 hours. ROS production was determined by DCFH-DA assay. JC-1 and TMRE were used to detect the mitochondrial membrane potential (A). The expression of cell stress related proteins was measured by western blotting after 24 hours of treatment (B). Data are presented as mean ± SD from three technical replicates. * p < 0.05, ** p < 0.01.

To examine the effects of IPAT and carboplatin-induced integrated cellular stress response, the ROS, JC-1, and TMRE assays were used in SPEC-2 and ARK-1 cell lines (Figure 3(A)). Measuring cellular stress *via* the production of intracellular ROS with DCFH-DA, treatment with the combination of 1 µM IPAT and 10 µM carboplatin for 16 h resulted in the largest relative ROS production in both SPEC-2 and ARK-1 cells. Treatment with either 1 µM IPAT or 10 µM carboplatin alone demonstrated an increased relative ROS production compared to the control in ARK-1 cells. Treating SPEC-2 cells with 1 µM IPAT resulted in a significant increase in the relative ROS production but treating with carboplatin 10 µM was no different compared to the control. JC-1 and TMRE fluorescence assays were used as inverse indicators of mitochondrial membrane potential (ΔΨm). Both assays demonstrated a significant reduction in ΔΨm when treating either ARK-1 or SPEC-2 cells with 1 µM IPAT and 10 µM carboplatin for 16 h. The combination of 1 µM IPAT and 10 µM carboplatin was more potent in reducing mitochondrial membrane potential than either IPAT or carboplatin alone in both cell lines.

The intracellular stress markers PERK and IRE1-α were assessed by western immunoblotting. Figure 3(B) shows an increase in both markers of intracellular stress when ARK-1 and SPEC-2 cells were treated with IPAT and carboplatin for 24 h, with the greatest effect seen in combination treatment compared to signal agent or control.

### Effect of IPAT in combination with carboplatin on DNA damage

**Figure 4.**
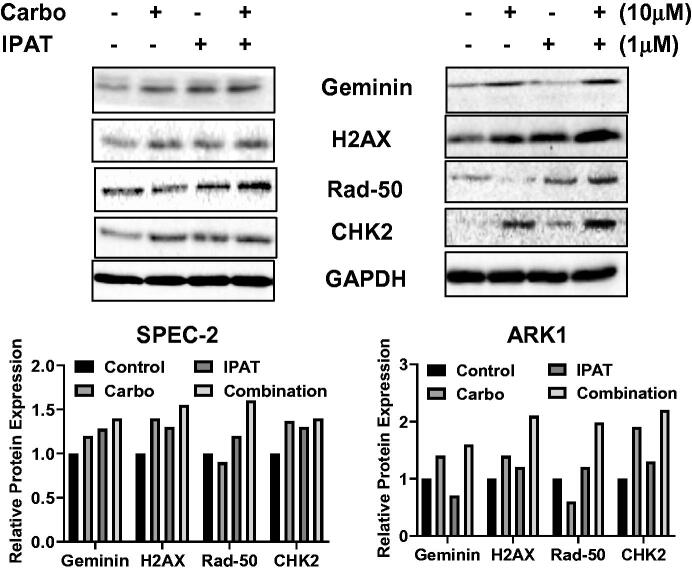
IPAT in combination with carboplatin induces DNA damage. SPEC-2 and ARK1 cells were treated with 1 uM IPAT, 10 uM carboplatin and their combination for 24 hours. DNA damage markers including geminin, r-H2AX, RAD50 and pCHK2 was detected by western blotting. All images shown here are representative of at least two independent experiments with similar results.

To clarify the combined effects of the IPAT combined with carboplatin on DNA damage, SPEC-2 and ARK-1 cells were treated with IPAT, carboplatin, or combination of both for 18–24 h. The results from western blotting demonstrated that carboplatin treatment increased the expression of Geminin, γ-H2AX, p-CHK2 and RAD50 in both cells, while IPAT also increased the expression of these proteins except for Geminin and γ-H2AX in the ARK-1 cells. The combination of IPAT and carboplatin revealed a more potent effect in most of these DNA damage markers (Figure 4). These results indicate that DNA damage pathways are involved in synergistic growth-inhibitory effects of combination therapy in USC cells.

### Effect of IPAT in combination with carboplatin on the invasion

**Figure 5.**
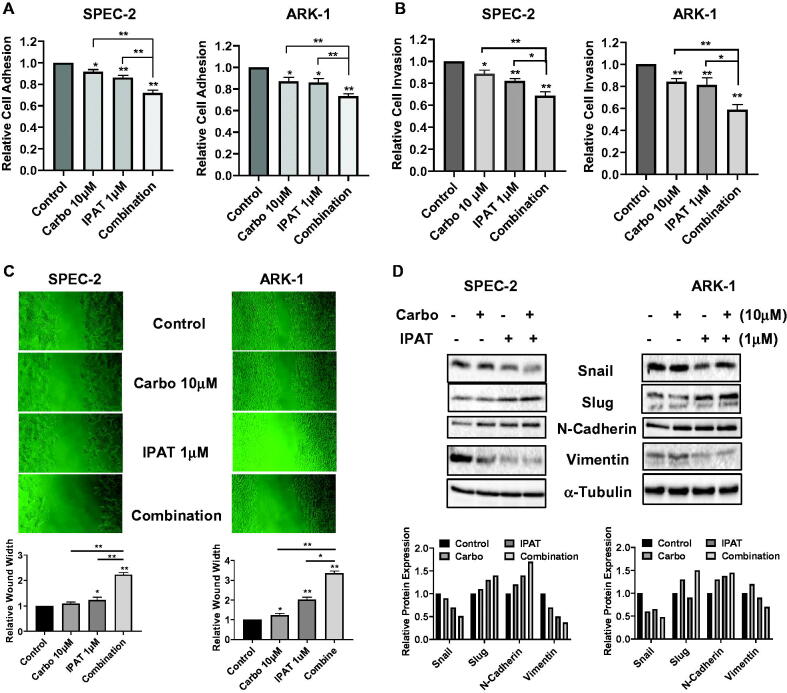
IPAT in combination with carboplatin reduces cell invasion. SPEC-2 and ARK1 cells were treated with IPAT (1 uM), carboplatin (10 uM) and their combinations for 4 hours, cell adhesion was determined by laminin adhesion assay (A). Invasion was measured using transwell assay and wound healing assay after 24-48 hours of treatment, respectively (B and C). Western blotting for EMT markers including Snail, Slug, N-Cadherin and Vimentin, in SPEC-2 and ARk1 cells, was done after the indicated treatments for 24 hours (D). Results are presented as the mean ± SD of three independent measurements * p < 0.05, ** p < 0.01.

To evaluate whether IPAT, carboplatin, and their combination inhibit migration and invasion in USC cells, the ARK-1 and SPEC-2 cells were incubated in laminin-coated 96-well plates and treated with IPAT, carboplatin and the drug combination for 4 h. Laminin adhesion assay demonstrated significant impairment in cellular adhesion in both SPEC-2 and ARK-1 cells after the treatments (Figure 5(A)). 1 µM IPAT inhibited cellular adhesion by 13.2% and 15.1% in SPEC-2 and ARK-1 cells, respectively, but 10 µM carboplatin alone only showed impaired adhesion in ARK1 cells. The combination of IPAT and carboplatin was significantly more effective in inhibiting adhesion than each of the single agents in both cell lines. Transwell assays also showed similar results for the inhibition of cell invasion in both cell lines, with combination treatment producing the greatest inhibition (Figure 5(B)). The anti-migratory activity of IPAT and carboplatin was evaluated by a wound-healing assay in both cell lines. The cells were treated with IPAT, carboplatin, or the combination at indicated doses for 24 and 48 h after wounding on the monolayer of cells. IPAT and carboplatin inhibited migratory potential in the wound healing assay by 89% and 22% in ARK-1, and 23% and 8% in SPEC-2 cells, respectively, with the combination of 1 µM IPAT and 10 µM carboplatin having the most pronounced effect (Figure 5(C)). Western blotting showed that IPAT decreased the expression of snail and vimentin, and increased the expression of slug and N-Cadherin, whereas carboplatin increased N-Cadherin expression and decreased vimentin expression in both cells. Combination treatments produced a more potent effect on the expression of vimentin, N-Cadherin, slug and snail after 24 h of treatment (Figure 5(D)).

## Discussion

Recent studies have found that the amplification of PIK3CA and mutations of PIK3CA and AKT are common molecular alterations and are involved in the aggressive biological behaviors of USC, suggesting that activation of the PI3K/AKT signaling pathway may be clinically relevant and targeting this pathway may be a particularly attractive strategy in order to turn off its oncogenic signaling [11,27]. Furthermore, the combination of anti-PI3K/AKT signaling agents with other conventional anti-tumor drugs may represent a new direction to overcome the intracellular escape mechanisms of conventional chemotherapies and increase chemosensitivity in patients with USC^16^[27,28]. Several reports have suggested that AKT inhibition by small molecular inhibitors may augment the efficacy of carboplatin in different cancer types. MK-2206, an allosteric AKT Inhibitor, showed synergistic responses in combination with carboplatin in lung and ovarian cancer cells while targeting Akt/mTOR axis *via* ABTL0812 significantly potentiated cisplatin anti-tumor activity in nude mice of lung cancer [29,30]. IPAT is a highly selective ATP-competitive pan-AKT inhibitor that inhibits AKT signaling to downstream biomarkers and has demonstrated potent anti-tumor activities in pre-clinical studies in multiple types of cancer and a phase II clinical trial of breast cancer [18,31]. In this study, we investigated the effects of IPAT, carboplatin and the combination on anti-proliferative and anti-metastatic activity in USC cell lines. The key findings are that IPAT in combination with carboplatin has synergistic efficacy in USC cells by inhibiting proliferation, enhancing cellular stress and apoptosis, inducing DNA damage, and reducing invasion. The combination of IPAT and carboplatin doses above IC50 values ​​exhibited CI values ​​of less than one over a wide range of concentrations in USC cells. High concentrations of IPAT combined with high concentrations of carboplatin did not display any synergy in the inhibition of cell growth, suggesting synergy between low-dose IPAT and low-dose carboplatin may be a more general phenomenon in USC cells.

The PI3K/AKT signaling pathway regulates survival and apoptosis by phosphorylation and inactivation of transcription factors and the primary regulators of the apoptosis cascade including p53, Bad, BCL-2, Bax, FOXO and NF-kB genes [32,33]. The BCL-2 family controls cell death evasion and resistance to therapy through rapid regulatory mechanisms [33,34]. Previous research studies have shown that IPAT inhibited cell viability through the induction of apoptosis in multiple types of cancer cells [18,35]. Moreover, a synergistic effect on apoptosis was observed with the combination of IPAT and other antineoplastic agents, including taselisib, paclitaxel, and duligotuzumab, suggesting that inhibition of PI3K/AKT signaling by IPAT is able to enhance the apoptotic response of combination treatments as compared to monotherapy in cancer cells [28,36]. We validated this observation with the combination of IPAT and carboplatin, as we observed a synergistic increase in cleaved caspase-3 levels in the IPAT and carboplatin combination in SPEC-2 and ARK-1 cells. In addition, we propose that increased apoptotic activity may be associated with the inhibition of pro-survival and anti-apoptotic proteins such as BCL-2 family proteins. This is supported by the fact that the combination treatments significantly reduced the expression of BCL-2 and MCL-1 in a synergistic fashion compared to single agent treatment in USC cells.

Aberrant PI3K/AKT signaling enhances cellular ROS levels through multiple molecular mechanisms, and increased cellular ROS in turn potentiates activation of PI3K/AKT signaling through inhibition of phosphatases or direct activation of oncogenes [37]. The accumulation of relatively large amounts of cellular ROS may directly cause DNA damage, activate apoptotic signaling and induce mutation [38,39]. Both IPAT and carboplatin has been shown to increase cellular ROS level and cause DNA damage in cancer cells [40–43]. Sensitivity of cancer cells to carboplatin involves increased activity of mTORC1/2 signaling and DNA damage and repair functions, and targeting AKT/mTOR pathway impairs DNA repair responses, resulting in increased chemosensitivity in a variety of cancers [44,45]. In the current study, we found a significant increase in ROS and a significant decrease in ΔΨm in USC cells exposed to IPAT. Combination treatment with IPAT and carboplatin produced a synergistic effect on ROS and ΔΨm compared with treatment with IPAT or carboplatin alone. Similar effects of IPAT in combination with carboplatin on DNA damage were also observed in SPEC-2 and ARK-1 cells, and combination treatment exhibited more potent induction on DNA damage and repair proteins including RAD50, CHK2, geminin and H2AX. Because RAD50 and H2AX are considered sensitive molecular markers of DNA damage and repair in response to carboplatin, we speculate that increased DNA damage may be the main mechanism by which IPAT increases the sensitivity of USC cells to carboplatin [46,47].

The PI3K/AKT pathway is implicated in regulating the migration, invasion and metastasis of cancer cells through acting on different downstream targets including initiating actin cytoskeletal rearrangements, cell polarization, remodeling the extracellular matrix (ECM) and induction of angiogenic cytokines [48,49]. Endometrial cancers with PIK3CA mutations have been associated with increased myometrial and lymphovascular invasion [50]. Inhibition of PI3K/AKT/mTOR signaling by miR-99a reduced cell invasion of EC, and MK2206, an allosteric AKT inhibitor, decreased tumor growth and invasion in patient-derived xenografts of EC [51,52]. IPAT in combination with either eribulin (antimicrotubule agent) or dasatinib (activated CDC42-associated kinase 1 inhibitor) significantly inhibited cell invasion in non-small-cell lung cancer and breast cancer cells [28,53]. Although IPAT or carboplatin alone induced a significant inhibition of adhesion in our findings, only treatment with the combination of IPAT and carboplatin was able to reduce invasive capacity in USC cells. Given the potential of the PI3K/AKT pathway to regulate cellular angiogenesis and invasion, it is reasonable to propose that inhibition of the PI3K/AKT pathway by IPAT serves as an enhanced mechanism for carboplatin to inhibit cell invasion.

Overall, these results suggest that the optimal antitumor effect of the combination of IPAT and carboplatin is mainly achieved by participating in the inhibition of the PI3K/AKT pathway, causing cellular stress and apoptosis, and inducing DNA damage in USC cells. To our knowledge, this is the first report to show inhibition of the PI3K/AKT signaling pathway by IPAT as well as synergizing and increasing sensitivity to carboplatin in USC cells. This has the potential for rapid translational impact as it provides a biological rationale for conducting clinical trials to evaluate the anti-tumor efficacy of the combination IPAT and carboplatin in patients with USC, cancer in desperate need of better treatment options.

## Data Availability

The raw data supporting the conclusions of this article will be made available by the authors, without undue reservation.
